# Adaptive Laboratory Evolution of Microorganisms: Methodology and Application for Bioproduction

**DOI:** 10.3390/microorganisms11010092

**Published:** 2022-12-29

**Authors:** Takashi Hirasawa, Tomoya Maeda

**Affiliations:** 1 School of Life Science and Technology, Tokyo Institute of Technology, 4259 Nagatsuta-cho, Midori-ku, Yokohama 226-8501, Japan; 2 Laboratory of Microbial Physiology, Research Faculty of Agriculture, Hokkaido University, Kita 9, Nishi 9, Kita-ku, Sapporo 060-8589, Japan; 3 Center for Biosystem Dynamics Research, RIKEN, 6-2-3 Furuedai, Suita 565-0874, Japan

**Keywords:** adaptive laboratory evolution, metabolic engineering, bioproduction

## Abstract

Adaptive laboratory evolution (ALE) is a useful experimental methodology for fundamental scientific research and industrial applications to create microbial cell factories. By using ALE, cells are adapted to the environment that researchers set based on their objectives through the serial transfer of cell populations in batch cultivations or continuous cultures and the fitness of the cells (i.e., cell growth) under such an environment increases. Then, omics analyses of the evolved mutants, including genome sequencing, transcriptome, proteome and metabolome analyses, are performed. It is expected that researchers can understand the evolutionary adaptation processes, and for industrial applications, researchers can create useful microorganisms that exhibit increased carbon source availability, stress tolerance, and production of target compounds based on omics analysis data. In this review article, the methodologies for ALE in microorganisms are introduced. Moreover, the application of ALE for the creation of useful microorganisms as cell factories has also been introduced.

## 1. Introduction

In bioproduction using microorganisms as cell factories, the efficiency of creating host cells is one of the key issues. Since the late 1990s, the genome sequences of various microorganisms have been determined, and various genome-wide analysis systems have been developed. The utilization of such genome-wide data is of great importance in the creation of microbial cell factories. In the 2010s, the development of next-generation sequencing technologies facilitated such trends in the field of bioproduction using microbial cell factories. Moreover, many researchers have performed adaptive laboratory evolution (ALE) to create microbial cell factories.

ALE can be defined as an experimental technique to facilitate cells to increase fitness to specific environmental conditions, which is established by researchers, through long-term cultivation [1]. Generally, ALE is a process in the laboratory based on the evolution of organisms, including the selection of species with effective mutations and/or recombination that can adapt or survive under certain environmental conditions. Analyzing the adaptation process in ALE enables not only an understanding of the adaptation processes of species but also the creation of microbial cell factories for bioproduction.

For ALE, repeated serial transfers of the culture to new media are conducted many times during batch culture. Cell growth is expected to increase through ALE as the fitness of the cells increases in a certain environment. ALE is conducted during chemostat cultivation as well, and an adapted cell population can remain in the culture. When cell fitness is increased, cell concentration increases during ALE using a chemostat.

The ALE experiment was initially reported in the 1950s; for example, Novick and Szilard reported the induction of spontaneous mutations during chemostat cultivation [2]. In 1988, Richard E. Lenski performed a long-term ALE experiment on *Escherichia coli* [3]. In this ALE experiment, 1% of 12 independent culture populations were transferred to a fresh medium every day and then incubated at 37 °C. Moreover, culture stocks were prepared at constant intervals for further analyses (details are described in Section 2.1). Surprisingly, Lenski and his colleagues have continued this process to date. Currently, the evolved *E. coli* cells have reached over 75,000 generations.

During ALE, cells acquire beneficial mutations to adapt to environments that researchers set, and as a result, changes in cellular physiological properties are induced, including the transcription process, translation process, metabolite synthesis, and metabolic fluxes. In order to understand cell adaptation processes in ALE, genome resequencing of the adapted population or isolated strains in the adapted population is performed to identify mutations that are responsible for increased fitness through ALE. Moreover, the transcriptome, proteome and metabolome of the adapted population or isolated strains from the adapted population are also analyzed. Based on genome resequencing and/or transcriptome data, effective mutations and/or changes in transcription, translation and metabolism that contribute to increased fitness via ALE can be identified. If necessary, the adapted strains are reconstructed based on such omics data.

At present, many methodologies for ALE have been established. For using ALE in bioproduction as well as fundamental research, it is important to determine which method(s) should be chosen for accomplishing the objectives. Moreover, ALE experiments, including environmental conditions, media, and microbial strains, have to be carefully designed considering the research objective(s) before starting ALE experiments because ALE experiments are based on long-term culture. In this review article, the ALE methodologies currently established are explained, and successful examples of studies on the industrial application of ALE for creating microbial cell factories toward bioproduction are introduced.

## 2. ALE Methods

**Figure 1:**
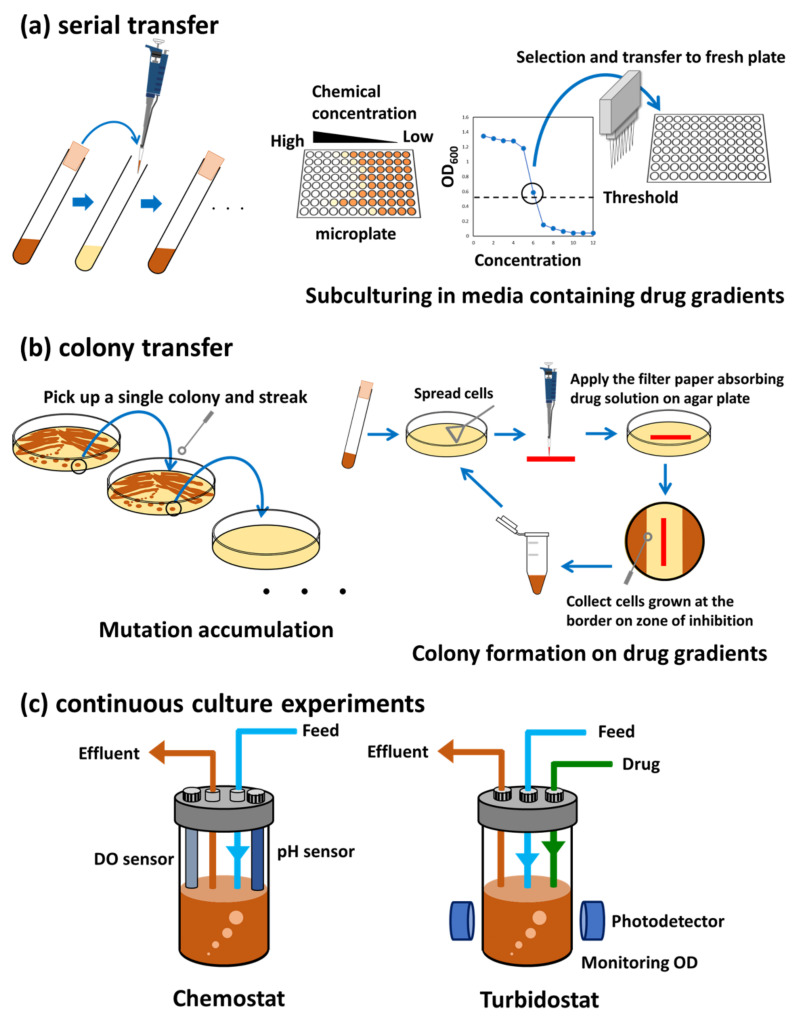
ALE methods. (**a**) Serial transfer. Serial transfer methods are based on repeats of subculturing on fresh media at regular intervals. For ALE linked to stress resistance, subculturing on media containing drug gradients is often conducted to monitor stress resistance and maintain the selection pressure during ALE. (**b**) Colony transfer. Mutation accumulation (MA) is a method to analyze the mutation rate and molecular spectrum of spontaneous mutations. MA protocol repeatedly picks up a single colony on an agar plate and streaks it out on the next fresh agar plate. In order to select the highest resistant cells during ALE of stress resistance, drug gradients can be generated on an agar plate by applying a filter paper absorbing drug solution on the agar plate. (**c**) Continuous culture experiments. Chemostat culturing using a jar fermenter can precisely control constant growth rates, population densities, nutrient supply, and environmental conditions such as pH and oxygenation. Instead of maintaining the constant substrate concentration, a turbidostat has feedback between the turbidity of the culture and the dilution rate.

ALE has been recognized as a promising methodology for improving microbial phenotypes and investigating evolutionary phenomena. In general, ALE can be categorized into three types of long-term culture methods; serial transfer, colony transfer, and continuous culture experiments (Figure 1 and Table 1).

### 2.1. Serial Transfer

Serial transfer methods are based on transferring an aliquot of the culture to a fresh medium at regular intervals for additional rounds of growth. In order to evaluate the reproducibility and/or stochasticity of evolutionary nature, several independent culture lines are usually propagated in parallel for each experiment. One of the most popular serial transfer-based ALE is the *E. coli* long-term evolution experiment (LTEE), which has been conducted by Dr. R. Lenski and his colleagues since February 1988 [3]. They started the LTEE using the 12 populations from the same *E. coli* B clone, and each independent 10 mL culture in a 50-mL Erlenmeyer flask was propagated daily by transferring 0.1 mL from the culture of the previous day into a fresh minimal medium containing 25 mg L^–1^ glucose as the limiting carbon source [3]. Samples were frozen at every 500 generations to assess and analyze time-course changes during the LTEE [4]. Various phenotypic changes have been reported during the LTEE, such as citrate utilization ability [5] and morphological changes [6]. *E. coli* can grow on citrate under anaerobic conditions when a co-substrate is available for reducing power [7], while it cannot transport citrate under aerobic conditions [8]. Although the evolution of the citrate utilization phenotype requires an extremely rare mutation, a citrate-using variant has evolved in one population by approximately 30,000 generations [5]. The dynamics of molecular evolution of the LTEE with *E. coli* over 60,000 generations confirmed that long-term adaptation to a constant environment is a complex process, with clonal interference, genetic drift, and eco-evolutionary feedback playing important roles [9].

Serial transfer-based ALE can also be applied to microbial co-cultures. For example, Konstantinidis et al. conducted ALE of obligatory mutualistic communities composed of wild-type lactic acid bacteria and engineered auxotrophic *Saccharomyces cerevisiae* to improve the secretion of metabolites with a high fitness cost [10]. In this study, *Lactobacillus plantarum*, which is an amino acid auxotroph producing and secreting vitamins (either riboflavin or folate) for the growth of vitamin auxotrophic *S. cerevisiae* mutant strains, whereas the *S. cerevisiae* mutant strain producing and secreting amino acids for the growth of *L. plantarum* [10]. The ALE of the two species started as the initial mixture containing both species in 1:1 volume, and stationary phase cells were repeatedly transferred 24 times corresponding to 160 generations with an initial optical density (OD) value of 0.01 at 600 nm [10]. After the ALE, the maximal OD values significantly increased and *L. plantarum* co-evolved with *S. cerevisiae* to improve vitamin secretion [10]. These results demonstrate that ALE of microbial communities based on mutualistic cross-feeding can be applied to increase the production of target compounds. Other examples of ALE based on mutualistic communities include the co-evolution of auxotrophic *E. coli* strains [11] and the co-evolution of auxotrophic *E. coli* and *Salmonella enterica* [12].

To analyze the adaptation mechanisms for antibiotics resistance and/or isolate antibiotics-resistant clones, ALE in the presence of high concentrations of antibiotics is applicable as a serial transfer method. Since the emergence of antibiotic-resistant bacteria is now considered a serious threat worldwide, ALE is often used to investigate the evolutionary dynamics of antibiotic resistance [13,14,15]. For these types of experiments, to increase the selection pressure during ALE, the antibiotic concentration is often increased concomitantly with the increase in antibiotic resistance of the evolved cells. For example, Lázár et al. conducted ALE of *E. coli* under 12 antibiotics with gradually increasing antibiotic dosage (1.5-fold increase) at every fourth transfer [16]. As an alternative method, cells can grow in media containing drug gradients, and the cells that can grow at the highest drug concentrations can be transferred to the next passages [17,18].

### 2.2. Colony Transfer

The colony transfer method is often used when the serial transfer of liquid culture is not applicable for ALE. Representative examples of colony transfer include the analysis of mutation accumulation (MA) used for the analysis of mutation rate and identification of the molecular spectrum of spontaneous mutations [19]. In contrast to ALE under certain selection pressures, the MA protocol is designed to accumulate unbiased mutations in a neutral way, devoid of selective pressure. Thus, the MA protocol repeatedly chooses a single colony on an agar plate and streaks it on the next fresh agar plates for thousands of generations. Picking single colonies on agar plates accomplishes the single-individual bottlenecks because almost all of the mutations that arise in a colony have no fitness effects. After the MA experiment, whole-genome sequencing of MA lines revealed an almost unbiased profile of mutations [19].

In the case of cells that form severe aggregates in liquid media, the colony transfer method can be an alternative experimental setup. For example, ALE of *Mycobacterium smegmatis* forming aggregates in ten anti-tuberculosis drugs was conducted on agar plates under various drug gradients [20]. In this experiment, the cells were first spread on an agar plate, and a filter paper soaked with the drug solution was placed on the agar plates. After incubation, cells at the border of the growth inhibitory zone were collected and deposited on a fresh agar plate with drug-soaked filter paper to increase the selection pressure. After three to eight repeats, evolved cells showing significant increases in drug resistance were isolated [20].

The colony transfer method can also be used for the ALE of co-culture. The discovery of a new antibiotic and its overproduction was achieved by ALE based on colony transfer [21]. In this study, the growth of an antibiotic-producing *Streptomyces clavuligerus* competed against that of methicillin-resistant *Staphylococcus aureus* (MRSA) on an agar plate and the grown *S. clavuligerus* was serially transferred until *S. clavuligerus* acquired the ability to constitutively produce the anti-MRSA drug, holmycin [21]. In this experiment, MRSA was spread onto an agar plate surrounding and in contact with *S. clavuligerus* completely to stimulate *S. clavuligerus* for the synthesis of the drug against MRSA. After incubation, colonies of *S. clavuligerus* that formed the largest zone of growth inhibition were isolated as single colonies. The isolated colonies were then subjected to the next passages for competition against MRSA. These steps were repeated until *S. clavuligerus* produced a significantly larger growth inhibition zone than that of the original parental clone [21].

Microbial evolutionary dynamics towards antibiotics can be directly visualized using soft agar, which allows bacteria to swim and spread [22]. Baym et al. developed the microbial evolution and growth arena (MAGA)-plate consisting of five successive areas containing black ink and different concentrations (gradually increasing towards the center of the plate) of antibiotics that are overlaid by thin, soft agar [22]. The time-lapse imaging of the MAGA-plate in which *E. coli* was inoculated could capture the real-time process of arising mutations and selections in the propagating front under exposure to high dosages of antibiotics [22].

### 2.3. Continuous Culture Experiments

The advantage of chemostat culturing using a jar fermenter is that it can control constant growth rates, population densities, nutrient supply, and environmental conditions, such as pH and oxygen concentration. However, a disadvantage of the chemostat methodology is that it is difficult to maintain multiple replicates in parallel owing to the costs of operation. In addition, cells can adapt to growing conditions in a bioreactor and may start forming biofilm to prevent washout from the reactor. Instead of a chemostat, a turbidostat that provides feedback between the turbidity of the culture and the dilution rate is also frequently used for ALE because an increase in fitness is often described as an increase in growth. Overall, the chemostat method is suitable for ALE, which requires strict control of environmental conditions for extended periods. For example, an *E. coli* strain that can synthesize sugars from CO_2_ was constructed by metabolic rewiring and chemostat-based ALE [23] (the details are described in Section 6.4).

**Table 1:** Three types of ALE methods.

ALE Method	Advantage	Disadvantage	Example of Applications
Serial transfer	Easy to automate and conduct high-throughput experiments	Not applicable to cells that aggregate or form biofilm in liquid culture. Growth is inherently discontinuous and control of the growth conditions is often limited and temporal.	*E. coli* long-term evolution experiment (LTEE) [3]. Co-cultures of obligatory mutualistic communities [10,11,12]. Resistance to chemicals [15,16,17]
Colony transfer	Introducing a single-cell bottleneck, applicable to aggregate cells in liquid media, and visualization of evolutionary dynamics using soft agar	Usually low-throughput and limitation to automation. Growth is inherently discontinuous and control of the growth conditions is difficult.	Mutation accumulation [19]. Anti-tuberculosis drug resistance of aggregating *Mycobacterium* [20]. Increase in antibiotics production by ALE of co-culture using *Streptomyces* and MRSA [21]. Visualization of antibiotic resistance using MAGA-plate [22].
Continuous culture experiments	It can control constant growth rates, population densities, nutrient supply, and environmental conditions	Limitation of multiple replicates in parallel owing to the costs of operation. Cells may adapt to bioreactors by the formation of biofilm to prevent washout	Morbidostat for antibiotic resistance [24]. ALE of *E. coli* strain that can synthesize sugars from CO_2_ [23]. Parallel turbidostat-based ALE of 78 *S. cerevisiae* populations by eVOLVER [25].

## 3. Automation Technologies for ALE

**Figure 2:**
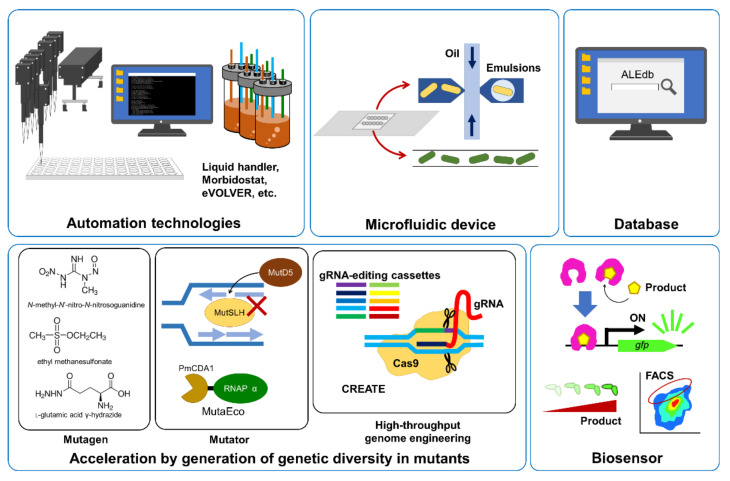
Useful tools for ALE. Automation technologies, including liquid handlers and automated culture control systems for continuous culture (morbidostat and eVOLVER) are powerful tools for ALE to deal with the limitations of manual approaches. To accelerate ALE, mutation rates can be increased by the addition of mutagens, exposure to UV, and manipulation of DNA replication or repair systems. High-throughput genome engineering, e.g., CREATE technology, can generate genetic diversity in mutants for ALE. For ALE to increase product formation, biosensors and FACS are useful to detect intracellular levels of product and sort cells showing higher titer. Microfluidic devices are highly manipulable, and they can be applied for the generation of precise chemical gradients in consecutive microcompartments. Additionally, emulsions such as “water-in-oil” microdroplets can compartmentalize cells inside the droplets. Thus, it can be used for the selection of beneficial cells with slow growth rates. Databases of mutations identified by many ALE studies are valuable for analysis and intuitive navigation.

Recently, many useful technologies and tools for ALE have been developed. From this section, such useful technologies and tools for ALE and some studies using them are introduced (Figure 2).

ALE experiments are often laborious because of the requirements of frequent measurements of cell growth and/or many independent culture lines as replicates for the evaluation of reproducibility. In the case of ALE for analyzing drug resistance in cells, cells are often inoculated in a drug gradient in several dilutions to monitor drug resistance and select the highest resistant population in each passage. ALE for improving growth rate under certain cultural conditions often requires to keep the cells in the cultures growing exponentially to select rapidly growing cells. It is very laborious to transfer cells frequently (e.g., at 2 to 6 h intervals) by researchers themselves. In addition, it has been reported that differing population sizes and selection pressures often affect the outcome of ALE [26]. In particular, selection pressures must be carefully adjusted because too strong selection pressure extinct benefit mutants while too weak selection pressure does not lead to evolution. Researchers need to achieve such precise control of conditions for ALE only with low throughput. Therefore, automation technologies have a high affinity for ALE to address the limitations of the manual approach. In addition to the increasing throughput, automation can avoid human errors and monitor the status in a short duration or in real-time, independent of the researcher’s schedule.

Automated liquid handlers are powerful automation tools for serial transfer-based ALE. They are automatically-pipetting robots that can precisely aspirate and dispense sample liquids in microplates and tubes. Some of the robots are equipped with robot arms to transfer plates to equipment to monitor cell growth, like microplate readers and shaker incubators. For example, Horinouchi et al. reported an automated culture system for ALE consisting of a Biomek NX span8 laboratory automation workstation (Beckman Coulter, Inc. Brea, CA, USA) in a clean booth connected to a microplate reader, shaker incubator, and microplate hotel [27]. By using the automated culture system, a high-throughput ALE of *E. coli* with the addition of 95 antibacterial chemicals was conducted for 27 d [17]. In this experiment, 576 independent culture series and 22 steps of drug gradients in 384-well microplates were maintained (95 drugs plus a control without any drug × 6 replicates) [17]. Lukačišinová et al. also developed a similar automated culture system and conducted an ALE of approximately 100 *E. coli* gene-deletion strains in the presence of antibiotics [26]. In this experiment, the cultures in 96-well microplates were incubated, measured the optical density (OD) every 10–15 min, diluted, and transferred to new plates, controlling both population size and selection pressure for drug resistance [26].

Chemostat- or turbidostat-based ALE can also be automated. For example, an automated fluidic system for continuous culture called ‘morbidostat’ was developed [24]. The morbidostat can constantly measure cell growth and adjust drug concentrations inside the culture vials to maintain the selection pressure as feedback [24]. The morbidostat comprises a flat-bottom glass vial, a magnetic stirrer for mixing the culture, an OD detection system, and a computer-controlled array of peristaltic pumps used for the liquid transfer [24]. In the case of the utilization of fluorescent reporters, a combination of fluorescence detection and a turbidostat called a ‘fluorostat’ could be a powerful tool [28]. A precise automated culture control system, eVOLVER, was developed for high-throughput automated culturing using a chemostat or turbidostat [25]. The system design is modular and synergistic, consisting of a fluidic manipulation system (peristaltic pumps or millifluidic devices), open-source software, and flexible hardware that integrates the sensors and actuators required to measure and control the parameters of individual cultures [25]. Parallel turbidostat-based ALE of 78 *S. cerevisiae* populations at distinct culture densities was conducted in glucose-limited media culturing for 500 h using the eVOLVER [25].

## 4. Acceleration of ALE

In some cases, the emergence of useful phenotypes takes a long time owing to the requirements of extremely rare mutations. In such cases, manipulation of the mutation rate by exposing cells to mutagens and introducing defective mutations in genes related to mismatch repair and proofreading can be a useful strategy for ALE [29,30]. Chemical mutagenesis by mutagens, including ethyl methanesulfonate and *N*-methyl-*N*’-nitro-*N*-nitrosoguanidine, is a classical method for screening desired mutant strains [31,32]. Historically, amino acid-producing strains used in industrial fermentations have been constructed by repeating mutagen treatments and selection cycles [33]. Recently, it was reported that L-glutamic acid γ-hydrazide (GAH) showed higher mutagenic activity than other known mutagens in *E. coli* [34]. The mutation rate of *E. coli* cells exposed to GAH was estimated as 0.59 per genome per generation, overwhelming the rate of the *E. coli* hypermutator strain Δ*mutL* [34]. Therefore, GAH may be applicable to the acceleration of the ALE.

Genes involved in DNA replication or repair systems are often modified to increase mutation rates. DNA polymerase III, which is the primary polymerase for bacterial DNA replication, has 3′ to 5′ proofreading exonuclease activity. Therefore, the mutant strains for the genes encoding DNA polymerase III (e.g., *dnaE* and *dnaQ*) can be mutator strains [35]. Furthermore, mutations in genes encoding the DNA mismatch repair (MMR) system also result in increased mutation rates since the fidelity of DNA replication is increased by MMR [19,36]. For example, the mutation rate of the *E. coli* ∆*mutL* strain, which is a strong mutator strain, was estimated as 1.3 × 10^−1^ per genome per generation, while that of the wild-type strain was 1 × 10^−3^ [19]. Such mutator strains have also been reported to emerge during long-term ALE populations [37]. For ALE, it is convenient to control the high mutation rates during the selection phase. For example, the overexpression of the DnaQ (also known as MutD) mutant protein (MutD5) derived from *E. coli* lacks catalytic activity, but retaining its binding ability to DNA polymerase III increases the mutation rate through competition with the functional DnaQ [38]. The use of a temperature-sensitive plasmid as the *mutD5* expression plasmid allows for the recovery of the wild-type level mutation rate in evolved strains [29]. Mutant variants resulting in mutator phenotypes have been widely used for ALE in *E. coli* and *S. cerevisiae* [29,39,40]. Treatments with mutagens or the introduction of mutations in genes encoding MMR and DNA polymerase III often cause cell damage or growth defects [41]. Recently, a new mutator protein (MutaEco) was developed to accelerate ALE without reducing cell viability [41]. MutaEco is a chimeric protein fused with a DNA-modifying cytidine deaminase and the α-subunit of *E. coli* RNA polymerase. Since the mutation rates are inversely correlated with cell viability, the mutation rate of the MutaEco-expressing strain is expected to be high enough to introduce sufficient diversification, but not too high to show toxicity [41].

High-throughput genome engineering techniques can be applied to constructing thousands of well-defined mutant libraries. Garst et al. developed CRISPR-enabled trackable genome engineering (CREATE) that can be applied to site saturation mutagenesis for protein engineering and genetically diverse libraries for ALE [42]. The CREATE method is a CRISPR/Cas9-based multiplex genome engineering integrated with barcode-enabled tracking of mutations in cell populations. CREATE uses modular guide RNA (gRNA)-editing cassettes comprising a gRNA sequence and a homology arm serving as a template for rescue from Cas9 gRNA-induced double-strand break. The cassettes can be designed in silico and synthesized ~10^6^ libraries on DNA microarrays. Then, large mutant libraries can be constructed by high-throughput cloning and transformation with Cas9 and lambda Red recombineering. Using the CREATE technology, ~10^5^ *E. coli* mutant libraries can be constructed by a single transformation experiment. Therefore, the CREATE technology can accelerate ALE by the generation of genetic diversity in mutants. Recently, Zheng et al. used the CREATE technology for constructing global regulator mutant libraries for ALE against furfural resulting in the obtainment of an evolved strain tolerated up to 4.7 g L^–1^ furfural [43].

## 5. Microfluidic Devices and Database for ALE

Microfluidic devices are useful tools for ALE because the flow rate of the media can be precisely controlled. However, microfluidics still has technical challenges; for example, preparation of spatial gradients for chemical concentrations and prevention of device clogging due to accumulated biomass. In order to overcome these technical challenges, Zoheir et al. developed a miniaturized fluidic chip for ALE that could generate precise chemical gradients in consecutive microcompartments [44]. Using the chip, culturing of *E. coli* for at least 14 days was achieved without clogging individual wells [44]. Interestingly, the chip-based ALE of *E. coli* identified novel mutations that confer nalidixic acid resistance [44]. MA microfluidic platforms can also be used for spatially segregated ALE. Emulsions such as “water-in-oil” microdroplets generated using microfluidics can compartmentalize cells inside the droplets [45]. Culturing in microdroplets is suitable for selecting beneficial mutants with slow growth rates; therefore, this methodology allows us to select for yield per droplet, not for growth rates [46]. A recent study reported the development of a microdroplet-based screening method for enhanced cellulase-producing *Pichia pastoris* based on a cellulase-catalyzed reaction that releases fluorescence [47]. 

As the number of studies on ALE has increased, a database of mutations identified by ALE could become a valuable tool for analysis and intuitive navigation. Currently, a web-based platform, “ALEdb,” storing the reported ALE-acquired mutations and their conditions from several model organisms is available (https://aledb.org/; accessed on 1 November 2022) [48].

## 6. Application of ALE to Improvement of Carbon Source Utilization in Microorganisms 

ALE can be used in studies on bioproduction using microorganisms as cell factories. In particular, improvement of carbon source utilization, creation of stress-tolerant microorganisms, and enhancement of productivity of target materials are the major goal of ALE for bioproduction. Hereafter, recent studies on the ALE of microorganisms considering bioproduction over recent years are introduced.

One of the important issues for bioproduction using microorganisms is the efficient utilization of carbon sources. The utilization of carbon sources derived from biomass resources (e.g., xylose) has been studied by many researchers. In addition, glycerol, which is a by-product in biodiesel production, and one-carbon (C1) compounds such as methanol, carbon monoxide (CO) and carbon dioxide (CO_2_) are realized as attractive carbon sources for bioproduction. ALE is an effective way to improve carbon source utilization and its modulation, because carbon source utilization is closely related to cell growth. Here, some significant studies on the improvement in carbon source utilization, such as xylose, glycerol, and one-carbon compounds, with ALE in microorganisms are reviewed.

### 6.1. Improvement of Xylose Utilization by ALE

Xylose is a five-carbon carbohydrate present in hemicellulose obtained from the hydrolysis of lignocellulosic biomass. Since hydrolysate of lignocellulosic biomass contains various carbohydrates, co-utilization of carbohydrates is important for efficient bioproduction. However, microorganisms such as *E. coli* and *S. cerevisiae* strictly regulate carbon source utilization. When glucose and other carbon sources are included in the culture medium, such microorganisms consume glucose first and then other carbon sources after depleting glucose. This mechanism is known as “carbon catabolite repression” or “diauxy.” Co-utilization of glucose and xylose is a target for the analysis of bioproduction using microorganisms. Improvement of xylose utilization and co-utilization of xylose with glucose using the ALE of microorganisms has been attempted. Recent studies on ALE of microorganisms for improvement of xylose utilization are summarized in Table 2.

### 6.2. Improvement of Glycerol Assimilation in S. cerevisiae with ALE

It is well known that glycerol is a by-product of biodiesel production, and its annual production reaches more than 750,000 tons. However, there is no effective way to utilize it. Since some microorganisms can assimilate glycerol as a carbon source, glycerol has become an attractive raw material for bioproduction using microorganisms as cell factories.

Although *S. cerevisiae* possesses glycerol catabolic metabolism consisting of the reactions catalyzed by glycerol kinase and glycerol-3-phosphate dehydrogenase, the glycerol assimilation ability of *S. cerevisiae* is very low [54]. For improvement of glycerol assimilation in *S. cerevisiae*, metabolic reactions responsible for glycerol assimilation, namely the dihydroxyacetone (DHA) pathway, need to be constructed in *S. cerevisiae*. As reported by Klein et al., the DHA pathway can be created by introducing the genes encoding glycerol dehydrogenase from a methylotrophic yeast *Ogataea parapolymorpha* together with overexpression of endogenous *DAK1* gene encoding dihydroxyacetone kinase and disruption of the *GUT1* gene encoding glycerol kinase [55]. To simply improve glycerol assimilation ability without constructing the DHA pathway in *S. cerevisiae*, ALE of *S. cerevisiae* grown on glycerol as the main carbon source would be efficient.

Strucko et al. reported that the ALE of *S. cerevisiae* on glycerol and increased fitness to conditions where *S. cerevisiae* can grow on glycerol was observed; growth on glycerol in the evolved population was 4-fold faster than that in the parental strain [56]. Genome sequencing of the evolved population, and genetic analysis suggested that the interaction of three causal mutations in *GUT1*, *KGD1*, and *UBC13*, which encode glycerol kinase, 2-oxoglutarate dehydrogenase, and a ubiquitin-conjugating enzyme, respectively, are important for efficient glycerol uptake.

In addition, Kawai et al. conducted ALE of *S. cerevisiae* using glycerol as the main carbon source and obtained evolved populations showing a specific growth rate of 0.14 h^–1^ after 85 generations, which is approximately three times faster than that in the unevolved parental strain [57]. Transcriptome analysis of the evolved populations during ALE revealed that the expression of the genes related to the TCA cycle was upregulated, and that of the genes related to the pentose phosphate pathway was downregulated. Based on these phenomena, they attempted to construct the recombinant strains of *S. cerevisiae* exhibiting high glycerol assimilation ability, resulting in the overexpression of the *HAP4* gene encoding a subunit of transcriptional activator complex for the TCA cycle genes or disruption of the *RIM15* gene encoding a great-wall protein kinase together with the overexpression of the *STL1* gene encoding a glycerol importer are effective for improving glycerol assimilation in *S. cerevisiae*. In addition, the metabolic flux distribution of the evolved strain was estimated using ^13^C-labeled glycerol [58]. However, the mechanism by which *RIM15* disruption improves glycerol assimilation ability remains unclear.

### 6.3. Creation of the Synthetic Methylotrophic E. coli, Which Can Grow on Methanol as the Sole Carbon Source with the Aid of ALE

Methanol is an attractive carbon source for bioproduction using microorganisms owing to its high electron content. Many methylotrophic microorganisms are present in nature, but information on the metabolism and genetic engineering tools for these methylotrophs is limited. Therefore, the creation of synthetic methylotrophs from *E. coli* and *Corynebacterium glutamicum* has been attempted by many researchers [59,60,61,62]. Synthetic methylotrophs are constructed by introducing metabolic reactions from methylotrophs for methanol oxidation [methanol dehydrogenase (Mdh)] and fixation of formaldehyde to ribulose-5-phosphate [3-hexulose-6-phosphate synthase (Hps) and 6-phospho-3-hexuloisomerase (Phi)], which is known as the formaldehyde fixation pathway and is called ribulose monophosphate (RuMP) cycle. Although these metabolic reactions are introduced into non-methylotrophic bacteria, their methanol assimilation ability is extremely low, probably because the supply of ribulose-5-phosphate (an acceptor in formaldehyde fixation) is limited.

It has been thought that the growth of synthetic methylotrophs on methanol as the sole carbon source is difficult. Recently, Chen et al. reported that synthetic methylotrophs of *E. coli* that can grow using methanol as the sole carbon source could be obtained with the aid of ALE [63]. The *rpiAB* disruptant of *E. coli* harboring heterologous Mdh and RuMP pathways could grow in the addition of xylose but not in xylose alone; disruption of the *rpiAB* gene was expected to increase the ribulose-5-phosphate supply for the RuMP pathway. They applied ALE to this recombinant strain to obtain a methanol auxotrophic mutant (i.e., increased methanol requirement for growth). In the evolved strain, the *pfkA* gene was further disrupted, and the *gapA* was replaced with *gapC* from another strain of *E. coli* to reduce the activity of glyceraldehyde-3-phosphate dehydrogenase and to remove metabolic instability. The *rpiA* gene was reintroduced, followed by ALE for 180 d to reduce the requirement of amino acids for growth, and as a result, the evolved strain that could grow on methanol without amino acid addition was obtained. Then, ALE of the evolved strain was further conducted on methanol plus nitrate, which can function as a terminal electron acceptor in the respiratory chain because of the richness of electrons in methanol. Finally, one methylotrophic strain of *E. coli*, that can use methanol as the sole carbon source, was obtained by single colony isolation from the evolved population. Genome sequence and transcriptome of the evolved synthetic methylotroph of *E. coli* revealed that an increase in copy number of the genes for Mdh and RuMP pathway mediated by insertion sequence is responsible and balancing of formaldehyde supply and consumption are responsible for efficient methanol assimilation. The authors concluded that the final methylotrophic strain exhibited a doubling time of 8.5 h, which was comparable to that of native methylotrophs. This is the first study to obtain the synthetic methylotroph of *E. coli* growing on methanol as the sole carbon source, and ALE should be essential to accomplish the objective.

### 6.4. Improvement of Growth of Synthetic Autotrophic E. coli on CO_2_ by ALE

The utilization of CO_2,_ as well as methanol, has also drawn attention as a good carbon source for bioproduction. Creating synthetic autotrophic microorganisms, which have the ability to fix CO_2_ to sugars, is challenging and has not yet been developed. However, Antonovsky et al. reported *E. coli* strains that can synthesize sugars from CO_2_ by introducing some of the heterologous reactions in the Calvin-Benson-Bassham cycle [phoshoribulokinase (Prk) and ribulose-1,5-bisphosphate carboxylase/oxygenase (RuBisCO)] [23]. To achieve this goal, they constructed a recombinant *E. coli* strain in which the CO_2_-fixing module with the upper part of the glycolysis and energy and reducing equivalent supply module, with the lower part of the glycolysis and TCA cycle working separately. In this strain, it was expected that by in silico metabolic simulation, CO_2_ fixation depended on xylose and pyruvate being served as a substrate for supplying ATP and reducing equivalents, but this strain could not grow on pyruvate, xylose, and CO_2_. Therefore, they attempted ALE so that this recombinant strain could grow on CO_2_ and pyruvate by conducting xylose-limited chemostat culture for approximately 2 months (approximately 150 generations). The evolved population grew on pyruvate and elevated CO_2_, with a doubling time of approximately 6 h.

The same group also attempted to use formate instead of pyruvate to supply energy and reducing equivalents for CO_2_ fixation in a constructed *E. coli* recombinant strain [64]. For this purpose, the heterologous formate dehydrogenase (Fdh) gene was introduced into the above recombinant strain. Formate was converted to CO_2_, and ATP and NADH were obtained. Similarly, the recombinant strain was subjected to ALE for approximately 350 d using a xylose-limited chemostat, and as a result, a chemo-organo-autotrophic *E. coli* strain was obtained. This evolved strain could grow on the CO_2_ produced from formate by the Fdh reaction. One of the isolated strains exhibited a doubling time of 18 h under autotrophic conditions. ^13^C-Labeling experiments confirmed that all carbon in the biomass was derived from CO_2_ in the evolved cells.

### 6.5. Improvement of CO Utilization by Acetogenic Bacterium with ALE

Acetogens are microorganisms that can convert CO, CO_2_ and H_2_, which are components of synthesis gas (syngas), to acetyl-CoA via the Wood-Ljungdahl pathway [65]. Acetogen can autotrophically grow on CO as a sole carbon source, but CO is very toxic. In syngas, 44% CO is contained, and it is difficult for acetogens to grow under such a high CO concentration. To obtain CO-tolerant acetogens, Kang et al. conducted ALE of an acetogen *Eubacterium limosum* under the conditions in the presence of 44% CO over 150 generations [66]. The growth of evolved strain exhibited a 1.44-fold increase in growth rate on 44% CO. Moreover, evolved populations can grow on more than 44% CO. The authors found that mutations in the *cooC* and *acsA* genes encoding CO dehydrogenase/acetyl-CoA synthase are causal. The evolved strain with an *acsA* mutation harboring acetoin biosynthesis genes could produce acetoin by 1.3-fold compared to the parental strain.

The same group performed ALE of the evolved *E. limosum* on 66% CO for about 400 generations [67]. Genome sequencing reveals that mutation in the acetyl-CoA synthase is responsible for increased CO conversion. Moreover, 2,3-butanediol synthesis pathways were implemented into the evolved strain and the resulting strain produced 2,3-butanediol in fed-batch culture on 66% CO. The production levels in the evolved strain were 6.5 times higher than those in the wild-type strain.

## 7. Acquisition of Stress Tolerance to Microorganisms with ALE

In bioproduction processes using microorganisms, host cells encounter various kinds of stresses derived from the culture environment, such as heat, osmotic pressure, oxidation, and pH change. Moreover, substrates for production and target products sometimes become toxic stress factors, such as methanol as a carbon source, ethanol and organic acids as production targets, and furfural and hydroxymethylfurfural as by-products of lignocellulosic biomass hydrolysis. These stress factors affect the activity of cells in the bioproduction processes, including growth, substrate consumption, and target production. Therefore, host microorganisms that exhibit tolerance to these stressors are desirable. To date, various reports on obtaining stress-tolerant microorganisms with ALE have been conducted on typical host microorganisms for bioproduction, such as *E. coli* and *S. cerevisiae* [68,69,70,71] (Table 3). In this section, impactful studies on stress-tolerant strains of other microorganisms obtained by ALE are introduced.

### 7.1. ALE for Conferring Stress Tolerance to Methylotroph with ALE

*Methylorubrum extorquens* (formerly named *Methylobacterium extorquens*), a methylotrophic bacterium, is a promising host microorganism for bioproduction using methanol as the carbon source. However, methanol is toxic to this bacterium, and even 1–5% methanol inhibits cell growth. Therefore, methanol-tolerant strains of *M. extorquens* are highly desired as bioproduction hosts. Cui et al. reported the breeding of a methanol-tolerant strain of *M. extroquens* via mutagenesis using atmospheric and room temperature plasma and ALE [72]. After eight generations of ALE, one mutant strain that could grow in the presence of 5% methanol was isolated. This strain exhibited seven times higher biomass yield than the parental strain, and the effectiveness of this strain for producing mevalonate in 5% methanol was validated. Belkhelfa et al. also obtained methanol-tolerant strains of *M. extorquens* by ALE using a turbidostat continuous culture system [73]. A mutant strain that could grow in the presence of 10% methanol was obtained after ALE for approximately 260 d. Genome resequencing analysis revealed that a mutation in the *metY* gene encoding *O*-acetylhomoserine sulfhydrylase, one of the substrates of methanol, is significant for methanol tolerance in the evolved strains.

### 7.2. Stress-Tolerant Strains of Non-Conventional Yeasts Obtained with ALE

The application of ALE has been expanded to various microorganisms including non-conventional yeast. *Torulaspora delbrueckii* is a yeast species related to wine brewing. *T. delbrueckii* can produce 7–9% ethanol and exhibits tolerance to that concentration of ethanol. Catrileo et al. applied ALE to *T. delbrueckii* to obtain strains showing tolerance to about 12% ethanol [74]. Serial transfers of culture with increasing ethanol concentration (6–12%) were performed for over 300 generations. One of the evolved clones showed a higher specific growth rate in the presence of 9% ethanol by 2-fold than the parental strain. Co-culture of the evolved *T. delbrueckii* with *S. cerevisiae* exhibited higher levels of total alcohols, total aldehyde, total phenolic compounds, and total sulfur compounds compared with the culture of *S. cerevisiae* alone.

Phommachan et al. reported ALE-evolved strains of *Candida tropicalis* showing tolerance to multiple stress [75]. They isolated the *C. tropicalis* strain in Laos, which is a thermotolerant xylose-fermenting yeast, but this species shows sensitivity to a high concentration of glucose. The authors performed ALE on a high concentration of glucose (200 g L^–1^) with gradually increasing culture temperature from 40 to 44.5 °C. After colony isolation from adapted populations, one adapted strain was obtained. This adapted strain showed increased tolerance to ethanol, furfural, and hydroxymethylfurfural at high temperatures and improved fermentation ability in the presence of a high concentration of glucose and xylose fermentation ability. The transcriptome of the evolved strain revealed upregulation of the genes related to glucose uptake and stress response.

### 7.3. Adaptation of Anaerobic Bacteria to Oxygen or Carbon Monoxide

Anaerobic bacteria are present in various anoxic and hypoxic environments. Because of their diversity and metabolic potential, those bacteria can be useful in industrial and environmental biotechnology. The application of anaerobic bacteria in biotechnology may require oxygen-insensitive hosts. The molecular mechanisms of oxygen toxicity to anaerobic bacteria and their defense systems against oxygen have not fully been elucidated yet [76]. Thus, ALE can be a powerful methodology to improve the adaptation of anaerobic bacteria to oxygen. Schoeffler et al. conducted oxygen-driven ALE of *Desulfovibrio vulgaris* Hildenborough, which is an anaerobic sulfate-reducing bacterium having the enzymes required for both sulfate and oxygen reduction [77]. The ALE of *D. vulgaris* was performed as successive cultures in Hungate tubes under continuous O_2_-sparging conditions at increasing O_2_ concentrations between 0.02% and 0.65%. After 114 generations, evolved strains showing increased oxygen tolerance and growth with oxygen reduction emerged. Further analysis revealed that optimization of redox balance (NADH/NAD^+^) accomplished the oxygen respiration in the evolved *D. vulgaris* [77].

Conversion of CO and syngas to industrially valuable products such as acetate and ethanol by anaerobic bacteria is thought to be promising. Fermentation of syngas by microbial consortiums rather than single species may be more suitable because of their resilience and the unnecessity of sterilization. Esquivel-Elizondo et al. conducted long-term ALE of microbial communities derived from anaerobic digester sludge was conducted with CO or CO and CO_2_ as the sole carbon and energy sources at incrementally increasing CO partial pressures [78]. The evolved communities showed increased production of acetate and ethanol from CO or syngas as the sole carbon and energy source [78].

### 7.4. High Light Tolerant Cyanobacteria Obtained with ALE

In photosynthetic microorganisms, as well as plants, intense light becomes a stressor and impairs photosynthesis. Cyanobacteria, photosynthetic microorganisms, are promising hosts for bioproduction using CO_2_ as a carbon source and solar energy. Intense light affects photosynthesis activity by damaging the photosynthesis machinery in cyanobacteria. Therefore, tolerance to light stress is an important characteristic of cyanobacteria bioproduction in open fields. Dann et al. obtained mutant strains of *Synechocystis* sp. PCC 6803, exhibiting tolerance to high light (2000 µmol m^–2^ s^–1^) by combining ALE with repeated mutagenesis using mutagens [79]. Although there are about 100 mutations in the tolerant strains associated with high light tolerance, they finally determined two important missense mutations in the *slr0844* (*ndhF*) and *sll1098* (*fusB*) encoding NAD(P)H-quinone oxidoreductase subunit F and elongation factor EF-G2, respectively. Moreover, Yoshikawa et al. reported a high-light-tolerant mutant of *Synechocystis* sp. PCC 6803 (9000 µmol m^–2^ s^–1^) was obtained using ALE alone for 52 d [80]. In the tolerant strain, excitation energy flow in photosystem II was maintained. Genome resequencing analysis revealed two mutations in *slr0484* (*hik26*) and *slr1916*, which encode a histidine kinase of a two-component signal transduction system and possible esterase, respectively.

## 8. Improvement of Production of Target Compounds by Microorganisms with ALE Integrated with Other Methodology

Methodologies for improving the production of target materials by microbial cells are highly desirable. ALE is an effective way to improve the production of target compounds using microorganisms. In particular, ALE is effective in improving the growth-coupled production of materials. If the growth of the host microorganism is improved by ALE, the production of the target material should be enhanced. For determining the target production to be enhanced by ALE, the relationship between metabolic pathways for producing the target and carbon catabolism to obtain energy for cell growth has to be considered. Recently, some studies on ALE for the improved production of target materials using microbial cells have been reported (Table 4). In this section, studies on the improvement of target material production using ALE, together with other methodologies such as co-culture and utilization of biosensors, are introduced.

### 8.1. Application of Co-Culture with ALE for Bioproduction by Microorganisms

Co-culturing of multiple microbial strains or species is a promising bioproduction system. Here, some studies on co-culture for bioproduction with ALE are introduced. In co-culture systems for bioproduction, the transfer of metabolites (produced by the donor and becomes the starting metabolite of the target compound produced by the recipient) from donor to recipient is important because the metabolite is diluted in the exterior of the cells before the recipient receives it. Therefore, both production of the transferred metabolite by the donor and its uptake by the recipient must be enhanced, and ALE is an effective method for this. Kawai et al. reported isoprenol production using a co-culture of *E. coli* recombinant strains and its enhancement by ALE [89]. In this study, the established co-culture of *E. coli* recombinant strains produced mevalonate from glucose and isoprenol from mevalonate, respectively. The latter strain required mevalonate for growth. To enhance the efficiency of mevalonate uptake in the mevalonate auxotrophic strain, ALE was used as the mevalonate concentration was decreased in a stepwise manner. Isoprenol synthesis pathways were introduced into the evolved strain and co-cultured with a mevalonate-producing *E. coli* strain. As a result, isoprenol production could be successfully improved compared with the co-culture of an unevolved isoprenol-producing strain with the mevalonate-producing strain.

The same research group also conducted ALE of a co-culture of leucine auxotrophic phenylalanine-producing strain with a phenylalanine auxotrophic strain of *E. coli* to improve phenylalanine production (i.e., mutualism) [90]. Subsequently, ALE was applied to this co-culture, and, as a result, an evolved strain of phenylalanine-producing strain was obtained.

### 8.2. Application of Biosensor Cells with ALE to Improved Bioproduction Using Microbial Cells

In many cases, ALE is linked to cell fitness, such as stress tolerance and nutrient utilization. However, this strategy does not always work for strain improvement in metabolic engineering, particularly for increased product formation. In order to overcome the limitations of ALE, biosensors are often useful tools for designing artificial selective pressures. Biosensor cells can detect intracellular metabolite levels in vivo. Briefly, in biosensor cells, a promoter sequence that works when the target gene is expressed in response to intracellular metabolite levels is connected to fluorescent protein genes such as the green fluorescent protein gene and the connected genes are introduced into the cells. If the target metabolite levels are increased in biosensor cells, the intracellular fluorescence increases. In the case of growth-coupled production of the target metabolite, ALE of the biosensor cells is an effective way to obtain mutant cells that produce high amounts of target metabolites. During the ALE, the fluorescence levels of the biosensor cells were monitored, and mutant cells that exhibited high fluorescence levels were obtained. Using such biosensors, cells that produce an increased amount of the product can be selected by fluorescence-activated cell sorting (FACS). Some examples of studies on ALE of microorganisms equipped with biosensors for improvement of the production of target materials are summarized in Table 5.

## 9. Conclusions and Future Prospective

To date, many researchers have conducted ALE experiments not only for fundamental studies but also for industrial applications in bioproduction using microbial cells as factories. In addition to the ALE studies described in this review, a study on ALE to obtain mutant enzymes that catalyzes metabolic reactions and the fluxes of their reactions coupled with cell growth were reported [95]. Furthermore, some studies on the application of ALE to overcome the problems in actual brewery processes using beer brewery yeast (for example, fermentation performance, flocculation, and undesired flavors) have been reported [96,97,98]. In order to achieve various objectives for bioproduction, a combination of ALE with other new methodologies is required.

As described in this review, there are many methodologies for ALE. Moreover, microbial species to be applied for ALE have been expanded. Considering the objective(s) for bioproduction using microbial cells, researchers need to choose the methodology of ALE. Moreover, to facilitate the rapid creation of microbial cell factories for bioproduction based on ALE, increasing the throughput of culture experiments and achieving reproducibility of experiments are both necessary for ALE. Automation of ALE experiments is required to accomplish this. Integration of various experimental systems will be highly desirable for ALE in the future. In addition, screening microbial strains with the desired phenotype(s) from cell populations obtained through ALE is a bit time-consuming. Therefore, the development of rapid screening methods for useful microbial strains, including culture experiments and genome-wide analyses, will be essential as well.

## Figures and Tables

**Table 2:** Recent studies on ALE for improved xylose utilization by microorganisms.

Organism/Strain	ALE Method	Results/Outcome	Reference
*E. coli* B with deletion of *ldhA*, *frdA* and *pflB* and replacement of *pdh* promoter with *gapA* promoter evolved on glucose and xylose, followed by *ptsG* deletion	Chemostat on glucose and xylose in bioreactor at a dilution rate of 0.01 h^–1^ for 7 days, followed by passages on agar plates containing xylose for 10 days and in Hungate tube containing liquid medium with stepwise increase in xylose concentration for 41 d	Adapted strain SCD78 simultaneously consumed both 10 g L^–1^ each of glucose and xylose in 36 h. High ethanol production was achieved.	[49]
*E. coli* MG1655 with deletion of *ptsH* and *ldhA* genes and replacement of *pflB* with *Z. mobilis pdc* and *adhB*	Serial transfers of culture on 10 g L^–1^ glucose for 5 times and on 6 g L^–1^ glucose and 4 g L^–1^ xylose for 5 times followed by transfers of cultures on 10 g L^–1^ glucose plus 5% ethanol	Adapted strain JK32E improved co-utilization of both glucose and xylose and produced high concentration of ethanol by additional introduction of the plasmid carrying *Z. mobilis pdc* and *adhB*.	[50]
*Z. mobilis* harboring the genes from *E. coli* related to xylose metabolism on the genome	Serial transfers of culture with increasing concentration of xylose from 3% (10 transfers), 5% (20 transfers), to 10% (20 transfers), followed by single colony isolations, for over 200 d in total.	The evolved strain AD50 showed increased xylose uptake rate by 1.65 times. Co-utilization of glucose and xylose and high ethanol production was achieved.	[51]
*Z. mobilis* carrying xylose isomerase gene from *Reticulitermes speratus* and *xylB* from *E. coli*	Serial transfers of culture containing 50 g L^–1^ xylose 38 times over 100 days.	Xylose consumption rate (50–150 g L^–1^) was increased and ethanol yield was enhanced by 60–140%	[52]
*Azotobacter vinelandii* OP	Serial transfers of culture on 2 g L^–1^ xylose and 8 g L^–1^ glucose and finally 8 g L^–1^ xylose and 2 g L^–1^ glucose in shake flasks (380 generations).	Specific growth rate, glucose uptake rate and xylose uptake rate in the evolved strain increase 2, 6.5 and 3.6-fold, respectively.	[53]

**Table 3:** Recent ALE studies for conferring stress tolerance to *E. coli* and *S. cerevisiae*.

Organism/Strain	Stress	ALE Method	Reference
*E. coli*	3-Hydroxypropionic acid	Serial transfers of culture in M9 medium containing 0.5 g L^–1^ yeast extract, 100 mM glycerol for 5 months. Concentration of 3-hydroxy propionic acid increased stepwise by 50 mM in the range from 200 to 800 mM	[68]
*S. cerevisiae* S288c	Thermal stress	Serial transfers of culture at 39.5 °C for 342 d (1200 generations)	[70]
*S. cerevisiae* and thermotolerant TTY23 strains	Acetic acid, acidic pH	Serial transfers of culture in the medium containing 3 g L^–1^ acetic acid at pH 3 for 41 d, 4 g L^–1^ for 57 d, and 12 g L^–1^ at pH 4 for 102 d (243 d and 816 generations in total)	[71]

**Table 4:** Recent studies on ALE of microorganisms toward improved production of target materials.

Target Product	Organism/Strain	ALE Method	Outcome	Reference
Recombinant protein	*Pichia pastoris*	Serial transfer of culture on methanol media for 250 generations.	One evolved clone using an alcohol oxidase (AOX) promoter for recombinant protein production showed 2.5- and 1.8-fold increased production in batch and fed-batch cultivations, respectively.	[81]
Itaconic acid	*Ustilago vetiveriae*	Serial transfer of culture on glycerol medium for 62 d.	Growth was not improved, while glycerol uptake was improved. Production of itaconic acid was improved, reaching 4.4 g L^–1^. Itaconic acid production by the evolved clone reached 35 g L^–1^ from 196 g L^–1^ glycerol with medium optimization.	[82]
Lactic acid	*Lactobacillus pentosus*	Serial transfers of anaerobic culture with increasing xylose concentration 100 times (850 generations).	Xylose consumption and lactic acid production increased by 1.5–2-fold compared to the parental strain on 20 g L^–1^ xylose.	[83]
Nonanedioic acid	*E. coli fadR* mutant	Serial transfers of culture on 3 g L^–1^ nonanoic acid 30 times (90 d).	The evolved strain with *fadE* deletion to prevent nonanoic acid degradation produced nonanedioic acid by 12.8-fold compared to the wild-type strain.	[84]
Glutaric acid	*C. glutamicum* recombinant strain for glutaric acid production applied to repeated cultivation	Serial culture transfers in glucose medium 8 times.	Growth was improved from 0.10 h^–1^ to 0.17 h^–1^. The volumetric productivity of the strain after the 7th transfer (0.18 g L^–1^ h^–1^) was two-fold higher than that of the starting strain.	[85]
γ-Aminobutyric acid	*E. coli* W engineered for γ-aminobutyric acid production from glycerol	Serial transfers of cultures in medium containing 0.2% glycerol for 1300 generations.	The specific growth rate was improved by 40%. Glycerol utilization was also improved. γ-aminobutyric acid production was increased by 2.6-fold.	[86]
Succinic acid	*E. coli* engineered for succinic acid production from glycerol	Serial transfers of culture on glycerol for about 100 generations.	Succinic acid yield from glycerol (C-mol/C-mol) was increased from 0.08 to 0.45.	[87]
Poly(3-hydroxybutyrate)	*Halomonas bluephagenesis*	Serial transfers of culture 71 times with increasing acetic acid concentration (20–120 g L^–1^).	Cell growth, acetic acid consumption, and tolerance to acetic acid were improved. Poly(3-hydroxybutyrate) production from acetic acid was improved as well.	[88]

**Table 5:** Biosensors used for ALE towards the increase in product formation.

Organism	Target Product	Biosensor	Outcome	Reference
*C. glutamicum*	L-Valine	L-Valine biosensor consists of *lrp*, the intergenic region between *lrp* and *brnF*, and a transcriptional fusion of *brnF* with *eyfp*.	Successfully evolved to a 25% increase in L-valine titers and a 3–4-fold reduction in by-product formation.	[91]
*E. coli*	Violacein	L-Tryptophan biosensor consists of *tnaC* fused with *tetA*-*sgfp*.	Successfully evolved to a 2.7-fold higher violacein production compared to the parent strain.	[92]
*C. glutamicum*	4-Hydroxyisoleucine	L-Lysine biosensor based on the transcriptional regulator LysG and EYFP.	Successfully evolved to a 28.4 % increase in 4-hydroxy isoleucine titers.	[93]
*E. coli*	3-Hydroxypropionic acid	3-Hydroxypropionic acid biosensor consists of two modules: the transcription factor C4-*lysR* sensing 3-hydroxy propionic acid, and the responsive module based on *tetA* expression conferring tetracycline resistance.	Archived production of 55 g L^–1^ 3-hydroxy propionic acid which is the highest yield reported to date.	[94]

## Data Availability

No new data were created or analyzed in this study. Data sharing is not applicable to this article.
